# Deprescribing as a Way to Reduce Inappropriate Use of Drugs for Overactive Bladder in Primary Care (DROP): Protocol for a Cluster Randomized Controlled Trial With an Embedded Explanatory Sequential Mixed Methods Study

**DOI:** 10.2196/56277

**Published:** 2024-07-23

**Authors:** Ann Lykkegaard Soerensen, Marie Haase Juhl, Marlene Lunddal Krogh, Mette Grønkjær, Jette Kolding Kristensen, Anne Estrup Olesen

**Affiliations:** 1 Department of Pharmacology Aalborg University Hospital Aalborg Denmark; 2 Department of Nursing University College of Northern Denmark Aalborg Denmark; 3 Department of Clinical Medicine Faculty of Medicine University of Aalborg Aalborg Denmark; 4 Department of Clinical Pharmacology Aarhus University Aarhus Denmark; 5 The Clinical Nursing Research Unit Aalborg University Hospital Aalborg Denmark; 6 Centre for General Practice Aalborg University Aalborg Denmark

**Keywords:** deprescribing, overactive bladder, general practice, patient safety, potentially inappropriate medication, geriatric, elderly, medication safety, geriatrics, anticholinergic drugs, safety, prescription, Denmark, general practitioner, evidence-based intervention, evidence-based, intervention, health care, medication, efficacy, DROP study, DROP

## Abstract

**Background:**

Potentially inappropriate medication remains a significant concern in general practices, particularly in the context of overactive bladder (OAB) treatment for individuals aged 65 years and older. This study focuses on the exploration of alternative options for treating OAB and the deprescribing of anticholinergic drugs commonly used in OAB. The research aims to comprehensively evaluate the efficiency of deprescribing through a mixed methods approach, combining quantitative assessment and qualitative exploration of perceptions, experiences, and potential barriers among patients and health care personnel.

**Objective:**

This study aims to evaluate the efficiency and safety of the intervention in which health care staff in primary care encourage patients to participate in deprescribing their drugs for OAB. In addition, we aim to identify factors contributing to or obstructing the deprescribing process that will drive more informed decisions in the field of deprescribing and support effective and safe treatment of patients.

**Methods:**

The drugs for overactive bladder in primary care (DROP) study uses a rigorous research design, using a randomized controlled trial (RCT) with an embedded sequential explanatory mixed methods approach. All general practices within the North Denmark Region will be paired based on the number of general practitioners (GPs) and urban or rural locations. The matched pairs will be randomized into intervention and control groups. The intervention group will receive an algorithm designed to guide the deprescribing of drugs for OAB, promoting appropriate medication use. Quantitative data will be collected from the RCT including data from Danish registries for prescription analysis. Qualitative data will be obtained through interviews and focus groups with GPs, staff members, and patients. Finally, the quantitative and qualitative findings are merged to understand deprescribing for OAB comprehensively. This integrated approach enhances insights and supports future intervention improvement.

**Results:**

The DROP study is currently in progress, with randomization of general practices underway. While they have not been invited to participate yet, they will be. The inclusion of GP practices is scheduled from December 2023 to April 2024. The follow-up period for each patient is 6 months. Results will be analyzed through an intention-to-treat analysis for the RCT and a thematic analysis for the qualitative component. Quantitative outcomes will focus on changes in prescriptions and symptoms, while the qualitative analysis will explore experiences and perceptions.

**Conclusions:**

The DROP study aims to provide an evidence-based intervention in primary care that ensures the deprescription of drugs for OAB when there is an unfavorable risk-benefit profile. The DROP study’s contribution lies in generating evidence for deprescribing practices and influencing best practices in health care.

**Trial Registration:**

ClinicalTrials.gov NCT06110975; https://clinicaltrials.gov/study/NCT06110975

**International Registered Report Identifier (IRRID):**

DERR1-10.2196/56277

## Introduction

### Background

Potentially inappropriate medication (PIM) is a significant issue in general practices. PIMs are defined as medications that may have an unfavorable risk-benefit profile, and alternative options may be better, safer, or more cost-effective [1-3]. Anticholinergic drugs for overactive bladder (OAB) are a common example of a PIM in older adults and are frequently used in general practice [4]. OAB is a chronic condition characterized by a frequent urge to urinate and nocturia, with or without urgency urinary incontinence [5]. Although available treatment options may improve symptoms, they do not cure them. Therefore, informing patients on therapeutic options is critical in general practice [6].

There are 2 main classes of drugs used to treat OAB: anticholinergic medications and selective beta-3 adrenergic agonist medications. The initial step in treatment involves nondrug methods, followed by the consideration of anticholinergic drugs. In cases where these drugs cannot be used due to medical reasons or prove ineffective, the option of using a selective beta-3 adrenergic agonist comes into play [7]. However, there is no consistent evidence to show the superiority of drug therapy over conservative therapy [8]. This lack of evidence emphasizes the need for general practice to consider the risks and benefits of treatment options carefully.

It is known that approximately 20% of patients treated with anticholinergic drugs experience adverse drug effects consisting of dry mouth and constipation. Older people can also experience impaired attention, delayed memory, visual disturbances, dizziness, and somnolence [9]. In addition to the potential adverse effects of anticholinergic drugs, general practitioners (GPs) must also be mindful of the risks associated with polypharmacy in older patients. Many older patients are already taking various medications for other conditions, and interaction with other drugs (eg, antidepressants, antihistaminic agents, and medication for Parkinson disease) leads to an increased risk of anticholinergic side effects [9,10]. Additionally, drugs with anticholinergic effects may be associated with a higher risk of falls [11] and an increased mortality rate among older adults [12,13].

International guidelines and the Danish Health Services recommend an early review of the efficacy and tolerability of drugs for OAB and a review and deprescribing once a year to assess continued needs [7,14,15]. In this study, “deprescribing” refers to the planned and supervised process of reducing or stopping medications [16]. Primary care, as the central stakeholder in the patient’s medication management, is typically the optimal setting for conducting medication reviews and deprescribing interventions.

In Denmark, when patients present with OAB symptoms, GPs play a pivotal role. They conduct medical histories, physical examinations, and basic tests to confirm the diagnosis. Specialists, such as urologists and urogynecologists, provide in-depth evaluations based on individual needs, and specialist consultations are required for advanced diagnostics and specialized treatment in complex cases with underlying pathologies. Both GPs and specialists can initiate pharmacological treatment for OAB. However, after initiating and ending treatment in specialist care, patients often return to their GP for follow-up.

However, several challenges at the patient level, the health care professional level, and the surrounding health care system may impact the success of deprescribing interventions [17]. Health care professionals report time constraints, limited data on the deprescribing process, and the need for feasible and effective medication management models to implement in routine care [17-19]. Ideally, the individual GP and the staff members should attain a sense of influence and ownership [18].

Deprescribing is an established management strategy to minimize polypharmacy and PIMs. It is, in general, a safe process, with a minor risk of causing withdrawal symptoms or return of the condition that was being treated [18,20]. Deprescribing is proven effective, leading to less treatment burden, reduced side effects, and lower medication expenses. However, there is less evidence on the impact of deprescribing on clinical and patient-centered outcomes [21]. Deprescribing is thus a multifaceted topic and requires a comprehensive understanding of both quantitative and qualitative aspects to deliver robust knowledge on the subject.

While several studies have investigated deprescribing by reviewing the entire medication list, focusing on a specific class of medicine can also effectively reduce adverse drug effects and improve quality of life. To our knowledge, no studies have investigated deprescribing interventions in primary care focusing on anticholinergic drugs for OAB. However, a recent quasi-experimental study conducted in a primary care setting investigated the efficacy of medication revision specifically for mirabegron. The study found that deprescribing optimized the use of mirabegron in 56.8% of cases [22], highlighting the potential benefits of a targeted deprescribing approach. By focusing on specific drug classes, GPs may be able to identify and address inappropriate prescriptions, leading to improved patient outcomes more effectively. Moreover, there is sparse knowledge on how health care staff and patients engage in the process of deprescribing drugs. Therefore, we aim to evaluate the efficiency and safety of the intervention in which health care staff in primary care encourage patients aged 65 years or older to participate in deprescribing their drugs for OAB. In addition, we aim to identify factors contributing to or obstructing the deprescribing process, which will drive more informed decisions in the field of deprescribing and support effective and safe treatment of patients.

### Objectives

The aim is divided into four objectives: (1) to evaluate the difference in the proportion of prescriptions picked up between the control and intervention groups; this metric will indicate the extent to which medication reduction has been achieved within the intervention group; (2) to evaluate the efficiency of the intervention regarding the proportion of patients in whom medication for OAB is deprescribed within the intervention group and the impact on symptoms; (3) to gain insight into the perceptions of patients and health care personnel regarding the deprescribing intervention by exploring their experiences and attitudes and identifying any potential barriers or contextual factors that may hinder future implementation; and (4) to comprehensively explore the complex phenomena of reducing the risk of potentially inappropriate drugs in general practice in patients aged 65 years and older with a specific focus on drugs for OAB.

## Methods

### Study Design

**Figure 1:**
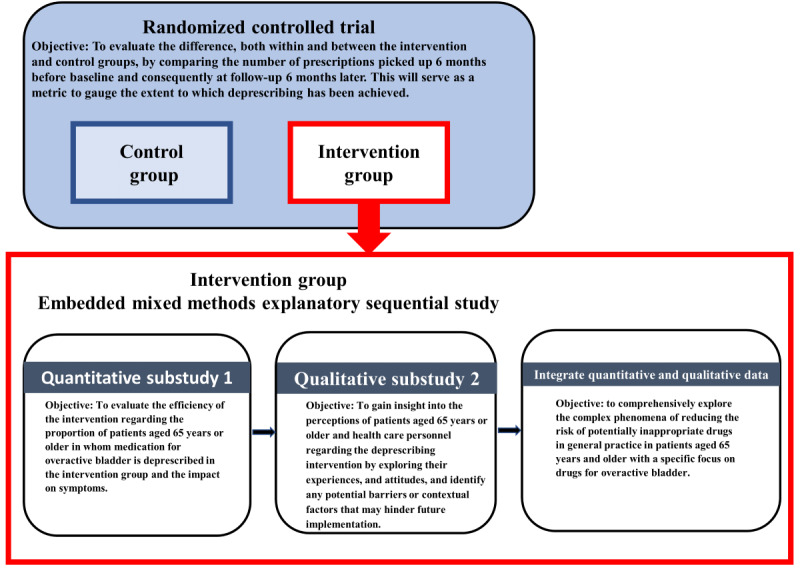
Overview of the drugs for overactive bladder in primary care (DROP) study. The intervention group, depicted by the red boxes, will provide data for the sequential explanatory mixed methods study.

The drugs for overactive bladder in primary care (DROP) study is a randomized controlled trial (RCT) with an embedded sequential explanatory mixed methods approach targeting the RCT’s intervention group. In the embedded mixed methods study, which will be carried out at the immediate conclusion of the RCT, quantitative data from the intervention group in the RCT and qualitative data from interviews and focus groups will be used to explore the nuances of how, when, and under which circumstances these individuals actively participate in the deprescribing process. Figure 1 provides an overview of the phases of the DROP study. Overall, this research design combines quantitative and qualitative data collection and analysis techniques to better understand the contextual factors influencing engagement in the deprescribing process among GPs, staff members, and patients.

The RCT will commence by pairing all general practices within the North Denmark Region, encompassing an estimated population of 0.58 million individuals. The pairing will be determined based on the number of GPs in each practice and their respective urban or rural locations. The matched pairs will be randomized to either intervention or control groups.

**Figure 2:**
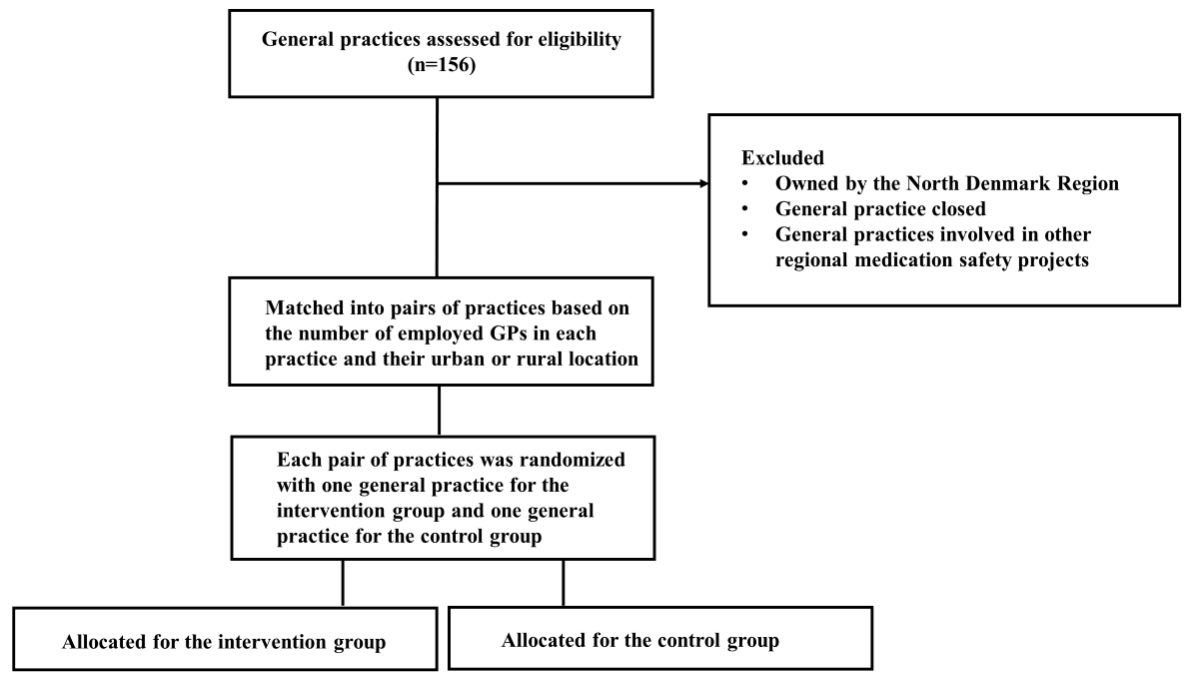
Flowchart illustrating the randomization process. GP: general practitioner.

The randomization for selecting these specific practices will be generated using the web-based tool “Research Randomizer” [23]. Figure 2 provides an overview of the matching and randomization process.

The intervention group will be provided with an algorithm designed to guide the deprescribing of drugs for OAB, aiming to promote the appropriate use of these medications.

### Randomized Controlled Trial

#### Overview

Using data from Danish registries, the RCT will evaluate the difference, both within and between the intervention and control groups, by comparing the number of prescriptions picked up 6 months before baseline and consequently at follow-up at 6 months. The 6-month follow-up period was chosen to balance practical considerations and the need for adequate evaluation time. The comparison to a randomized control group will allow for an assessment of whether other external factors have impacted the results using safety measures. The proportion of prescriptions picked up between the control and intervention groups does not directly measure deprescribing as defined by discontinuing and reducing dosage. Instead, it serves as an indicator of potential changes in medication use.

#### Primary Outcome

The primary outcome will be the difference in prescriptions picked up 6 months before the baseline and 6 months after the baseline.

#### Secondary Outcomes

The secondary outcomes will be the descriptive characteristics of the study population and the difference in hospital admissions, health care contacts, and mortality between the intervention and control groups.

### Embedded Sequential Explanatory Mixed Methods Study

This part of the DROP study will support a deeper understanding and explanation of the results of the RCT.

#### Quantitative Substudy 1: Follow-Up in the Intervention Group

##### Overview

Substudy 1 will estimate the proportion of patients who discontinue drugs for OAB or reduce the dosage of OAB with a 6-month follow-up. Staff members will fill out a baseline questionnaire and provide descriptive data about the cohort. A total of 2 questionnaires about symptoms at 4 weeks and 6 months will be filled out using self-reported data. The descriptive data will encompass significant variables like family status and other concurrent illnesses.

##### Primary Outcome

The primary outcome will be the proportion of patients who were deprescribed OAB before and after a 6-month follow-up period.

##### Secondary Outcomes

The secondary outcomes will be as follows: changes in bladder symptoms, descriptive characteristics of the proportion of patients in the intervention group who had deprescribing initiated and sustained over a 6-month follow-up period, descriptive characteristics of the proportion of patients in the intervention group who had deprescribing initiated but not sustained, and descriptive characteristics of the proportion of patients in the intervention group where deprescribing was not initiated.

#### Qualitative Substudy 2: Interviews and Focus Groups

Substudy 2 will use qualitative interviews and focus groups to explore how GPs, staff members, and patients experienced the intervention and which strategies have been used to successfully deprescribe OAB to understand when and how the algorithm is applicable. Participants will possess a variety of criteria informed by the quantitative data to ensure maximum variation and will be selected based on purposeful sampling. Interview guides will be developed based on the existing literature.

### Integration and Interpretation

Subsequently, the quantitative and qualitative results will be integrated, describing congruence and complementarity or the lack thereof. Finally, an interpretation of the integrated data will form inference as part of the answer to the overall aim.

Integration of the 2 types of data sets occurs at multiple stages in this study. First, integration takes place during the qualitative interview sampling process. Second, integration is evident in developing semistructured interview guides informed by the quantitative results. Finally, integration is vital to the analysis phase, where joint display methods will be used.

Through a merging process, we combine the quantitative results from the follow-up study on deprescribing OAB and the qualitative data gathered from interviews with GPs, staff members, and patients. We aim to interpret these integrated data collaboratively. Specifically, we focus on how health professionals and patients articulate their experiences with deprescribing treatment for OAB, their interactions with the algorithm, and any advantages or shortcomings they have identified during its extended use. Additionally, we seek to ascertain if circumstances or life events critically influence patients’ ability to complete the deprescribing process.

### Study Setting

The study will take place in The North Denmark Region, Denmark. In Denmark, GPs contract with and are reimbursed by the National Health Service. Traditionally, GPs practice singly, but there is a tendency to join groups of practitioners. All Danish residents are entitled to register with a GP of their own choice, and >99% of the population are registered with a GP, and on average, each GP serves 1600 patients. Though no clear guidelines exist for when and how to treat the individual patient, the GP is advised to evaluate the pharmacological treatment for OAB yearly by suggesting the patient discontinue the drug for approximately 4 weeks [24].

### Participants

#### GP Practices

All GP practices in The North Denmark Region, Denmark, will be eligible for randomization. It is important to note that GP practices already participating in interventions focusing on medication reviews will be excluded from the study before the randomization, as this could potentially interfere with the outcomes in both the intervention and control groups. There are currently 156 practices in the North Denmark region, of which half will be invited to participate. GP practices randomized to the intervention group will receive an initial invitation via email, followed by subsequent telephone contact. Those GP practices that accept the invitation and agree to participate will be enrolled in the study. As a token of appreciation for their involvement, these practices will receive a small reimbursement for their participation in the study.

#### Patient Recruitment

A list of patients (65 years or older), who, within the last 14 months, have been prescribed a drug for OAB by a GP will be generated and presented to the included GP practices. Table 1 provides a list of the relevant drugs. Staff members will then identify the patients in the GP practice’s electronic system. Patients receiving outpatient care for OAB (eg, in urological or neurological clinics) or are cognitively impaired to a degree incompatible with participation will be excluded.

**Table 1:** Overview of drugs included in this study.

Anatomical Therapeutic Chemical code	Name
G04BD04	Oxybutynin
G04BD07	Tolterodine
G04BD08	Solifenacine
G04BD09	Trospium
G04BD10	Darifenacin
G04BD11	Fesoterodine
G04BD12	Mirabegron

#### Sample Size

According to an unpublished pilot project, approximately 26% of patients in current treatment may be candidates for deprescribing drugs for OAB. With a 5% margin of error, 95% CI, and a population of 500 patients (for the GP practices randomized to the intervention), it was calculated that 187 patients should participate in the study. Therefore, 250 patients will participate to give room for “dropouts” and “missing data.” Control patients will consist of a similar number of patients in the control group who are currently treated. The mixed methods study does not report a desired sample size as emphasis is placed on the richness and depth of collected data by integrating qualitative and quantitative data. Instead, we aim to ensure an adequate range of patients and staff to capture diverse perspectives and experiences. The focus is on the data’s quality, relevance, and credibility rather than a predetermined numerical sample size [25].

#### Deprescribing Intervention

The clinical guideline on “Deprescribing drugs for OAB” in The North Denmark Region serves as the basis for the intervention. The guideline, originally published in Danish, provides recommended procedures for the deprescribing process. The guideline offers procedures for the deprescribing process and includes a deprescribing algorithm and a symptom questionnaire designed to evaluate the impact of medications used in treating OAB [26]. The questionnaire consists of inquiries about the patient’s bladder symptoms such as how they affect their daily life and whether there are any medication-related side effects. The intervention encompasses a systematic approach to support GPs, staff members, and patients in evaluating the feasibility of discontinuing medication. This systematic approach, bolstered by support from the research group, goes beyond merely presenting the guideline; it aims to facilitate its systematic implementation within clinical practice. The research group’s involvement is geared toward evaluating the guideline’s efficacy and identifying optimal implementation strategies, all while ensuring patient safety and minimizing practitioner effort. It is important to note that clinics already using similar routines are still eligible for participation in this study. Practically, staff members will contact each patient for whom deprescribing may be relevant and gather baseline characteristics over the phone. Patients who agree to participate in the deprescribing intervention can provide their data web-based or via phone call with the GP practice. Whether the questionnaire is filled out by the patients themselves or the staff members, all the data will be securely stored in REDCap (Research Electronic Data Capture; Vanderbilt University) [27]. REDCap is a secure web application that builds and manages web-based surveys and questionnaires. The patient’s treatment is then deprescribed, and 4 weeks later, the patient evaluates the effect either web-based or by receiving a phone call from the GP practice, using a symptom questionnaire, and following the algorithm described in clinical guidelines. The final follow-up, using the symptom questionnaire, will take place 6 months after deprescribing.

#### Timeline

The study, in which GP practices are invited to participate, launches on December 1, 2023.

### Methods of Data Collection

#### Randomized Controlled Trial

In the RCT, we will apply solely registry data to evaluate the efficiency of the intervention. The intervention and the control group will be compared using registry data including prescription records, sociodemographic information, and health-related outcomes such as hospitalizations and mortality rates. The data will be extracted from the appropriate Danish registries. It will be essential to know whether the algorithm is effective and identify potential safety issues, such as higher hospitalization or mortality rates.

#### Embedded Sequential Explanatory Mixed Methods Study

The mixed methods part of the study consists of quantitative data from substudy 1, which is the follow-up in the intervention group, and qualitative data from substudy 2, which is generated from interviews and focus groups.

#### Substudy 1: Follow-Up in the Intervention Group

Substudy 1 will estimate the proportion of patients who can discontinue OAB or reduce the dosage of OAB with a 6-month follow-up considering bladder symptoms and patient characteristics. See the description for the deprescribing intervention.

#### Substudy 2: Qualitative Interviews and Focus Groups

Once substudy 1 is completed, we will conduct qualitative in-depth interviews at 4 weeks and 6 months to explore the experiences of GPs, staff members, and patients regarding deprescribing drugs used to treat OAB. Individual qualitative interviews and focus groups were deemed suitable and valuable for capturing the perceptions and experiences of the intervention and deprescribing medication [28]. By using individual qualitative interviews, it is possible to explore and clarify experiences and opinions with as much information as possible and with the participant’s own words and voice [28]. Interviewee selection will be based on purposeful sampling, an iterative process that aims to achieve richness and depth in data [28]. Participants will be sampled based on variation in role (GPs, staff, or patients), clinic size, gender, age, and location, divided into urban and rural. If possible, we will sample one GP, one of the staff, and one patient from 8 different clinics as we believe this to meet the aim of this study, sample specificity, theory, anticipated quality of the dialogue, and the applied analysis strategy [29]. However, this will be applied should elements of information power [29] call for a change in strategy. Information power feeds into data saturation, which will be achieved by using the model of Guest et al [30] model, incorporating elements such as Base Size, Run Length, and New Information Threshold to determine saturation points in the data collection. The interviews will be planned and carried out based on the Theoretical Domains Framework “determinants of behavior” [31] and the 7 stages proposed by Brinkmann and Kvale [32].

### Methods of Data Analysis

#### Randomized Controlled Trial

Using an intention-to-treat analysis, an evaluation of the deprescribing intervention will be done by comparing primary and secondary outcomes between the intervention and control groups. Additionally, comparing variables at baseline will provide a basis for assessing the impact of the deprescribing intervention over time. Access to register data for both the intervention and the control group facilitates a sensitivity analysis by systematically analyzing participation and adjusting for potential confounding factors, thus providing a robust assessment of selection bias and enhancing the reliability of the study results. The distribution of variables will be evaluated using histograms and the Shapiro-Wilk test. Nonnormal data will be analyzed using boot-strapping resampling methods, and differences between groups will be examined using regression analysis. Binary outcomes will be compared using the chi-square test and reported as the number and percentage of cases. A *P* value less than .05 will be considered statistically significant.

#### Embedded Sequential Explanatory Mixed Methods Study

##### Substudy 1: Follow-Up Study

We will assess the efficacy of the deprescribing intervention in the intervention group by comparing primary and secondary outcomes at baseline and follow-up. Additionally, we will compare variables to evaluate the evolving impact of the deprescribing intervention over time. To understand the distribution of variables, we will use histograms. Differences between groups will be examined using regression analysis. Binary outcomes will be compared using the chi-square test, with results reported as the number and percentage of cases. We will consider a *P* value less than .05 as statistically significant.

##### Substudy 2: Qualitative Interviews and Focus Groups

The data will be analyzed using thematic analysis [33] to identify and explore key themes and patterns within the interview data. This will involve carefully reviewing and coding the data, followed by categorizing these codes to derive overarching themes. This approach will assist us in comprehending the perspectives and behaviors associated with deprescribing medication. We recognize that this field of study is novel and complex, requiring careful analysis and interpretation.

#### Integration and Interpretation

The design will include data sets from the quantitative analysis and evaluation of the study to establish the effect of the intervention longitudinally and a qualitative analysis of interviews with stakeholders (GPs, staff members, and patients) to understand the experiences and opinions that might influence the process of GPs. The 2 types of data sets will be analyzed separately before merging in final integration using a “joint display” [34]. The joint display will assist in understanding how the qualitative data supports the understanding of quantitative data and draw inferences [35], which may help in best practices when discontinuing relevant categories of drugs.

### Ethical Considerations

The study has received approval from the Institutional Management at Aalborg University Hospital. Under Danish legislation, no formal permission from the national or regional Committee on Health Research Ethics is needed for this type of study, as it does not involve treatments inferior to standard care or the collection of biological material. Thus, the North Denmark Region Committee on Health Research Ethics has reviewed the DROP study and deemed it exempt based on its design, which involves only surveys and interviews (2021-000438). This study will be carried out as a quality improvement project, thus specific data collection does not require informed consent. Nevertheless, informed consent will be obtained for all qualitative data. Participation in GP practices will be voluntary, and informed consent will be obtained from them. Patients’ participation is voluntary. Both GP practices and patients can withdraw from the study at any time. Danish legislation prohibits patient compensation for participating in research, but the participating general practices will be compensated for their time. The study complies with the General Data Protection Regulation and is part of the North Denmark Region's record of processing activities (K2023-012). It is also registered on ClinicalTrials.gov (NCT06110975). No identifying information will be published. The DROP study will adhere to the Declaration of Helsinki (64th WMA General Assembly, Fortaleza, Brazil, October, 2013.

## Results

The DROP study is ongoing. GP practices are randomized but not yet invited for participation. GP practices will be included from December 2023 to April 2024. The follow-up period for each patient is 6 months. Thus, the expected follow-up for the last patient is scheduled for November 2024.

## Discussion

### Principal Findings

The DROP study will generate a GP-led intervention, which is expected to lead to a reduction in the use of potentially inappropriate drugs. The overall quality of the intervention experienced by the patient will likely remain unchanged or improved. At the same time, doctors and support staff are anticipated to be satisfied with the process and the intervention. It is also expected that the methods used for the intervention are transferable to other drug classes. The study design combining several methods could improve research outcomes with the increasing complexity of the area under investigation, allowing for a deeper understanding of complex phenomena [35,36]. Thus, the RCT with an embedded integrative mixed methods analysis is expected to provide evidence for efficacy and a more comprehensive understanding of the deprescribing process, minimizing the risk of generating misleading results and guiding future clinical practice.

### Limitations

Systems- and organizational-related factors, such as political changes and resources, could be limitations in all stages of the DROP study. Moreover, GP engagement may fluctuate and diverge due to various factors. It is essential that GPs take ownership of the intervention, and the intervention must be simple and feasible in practice. Acknowledging the potential limitations of a 6-month follow-up period for data collection, our mixed methods approach will consider it valuable information for future studies of deprescribing in general practices. Although a possible limitation, the study will contribute to a broader understanding of the most appropriate follow-up duration in this context, enriching our insights for future research endeavors.

### Conclusions

The DROP study will generate evidence on interventions in primary care, ensuring that drugs for OAB are deprescribed when an unfavorable risk-benefit profile exists. This could lead to lower medicine costs for the patient and society, as there may be better, safer, or more cost-effective alternatives when the risk-benefit ratio favors deprescribing in people aged 65 years or older. Information drawn from the 2 approaches will support a generic model for deprescribing individual drug classes. Results from an RCT combined with insights from mixed methods research will prove instrumental in understanding why these results are achieved. This dual approach enhances the validity of our findings and offers a comprehensive view of the underlying mechanisms and contextual factors driving the observed outcomes. This comprehensive perspective is vital for generating robust results of deprescribing specific drug classes, thereby fortifying best practices in health care.

## Abbreviations

DROP: drugs for overactive bladder in primary care
GP: general practitioner
OAB: overactive bladder
PIM: potentially inappropriate medication
RCT: randomized controlled trial
REDCap: Research Electronic Data Capture

## Data Availability

The data sets generated or analyzed during this study are available from the corresponding author on reasonable request.
